# Refractive index and formaldehyde sensing with silver nanocubes†

**DOI:** 10.1039/d0ra10161c

**Published:** 2021-02-19

**Authors:** Hemant Ramakant Hegde, Santhosh Chidangil, Rajeev K. Sinha

**Affiliations:** Department of Atomic and Molecular Physics, Manipal Academy of Higher Education Manipal – 576104 Karnataka India rajeev.sinha@manipal.edu

## Abstract

We report the synthesis of Ag nanocubes by using a sodium sulfide assisted solvothermal method. Small edge-length nanocubes (32 and 44 nm) were obtained at 145 and 155 °C reaction temperature in the synthesis process. The refractive index sensitivity of synthesized nanocubes was investigated with an aqueous solution of glucose. The refractive index sensitivity of 161 nm per RIU was found in the colloidal dispersion of nanocubes. On the LSPR chip made by immobilization of nanocubes on the (3-aminopropyl)trimethoxysilane modified glass coverslip, the obtained sensitivity was 116 nm per RIU. Detection of formaldehyde in water and milk samples was also performed with nanocubes of edge-length of 44 nm. Formaldehyde detection was performed by utilizing the interaction of the aryl amine of 4-aminothiophenol immobilized on the nanocubes and electrophilic carbon atom of the formaldehyde. In water and in diluted milk, the formaldehyde sensitivity of 0.62 and 0.29 nm μM^−1^ was obtained, respectively.

## Abstract 2

A detailed description of refractive index and formaldehyde sensing with Ag nanocubes.

## Graphical abstract

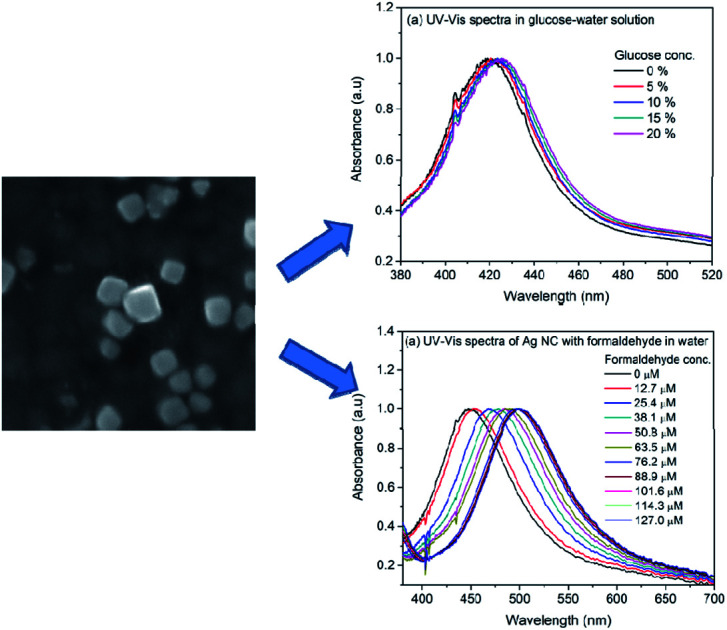

## 1. Introduction

Noble metal nanoparticles exhibit unique optical properties due to resonant oscillation of conduction electrons present on the surface, this phenomenon is known as localized surface plasmon resonance (LSPR).^1^ The LSPR peak position of plasmonic nanostructures is sensitive to the changes in the refractive index of the surrounding medium.^2^ Anisotropic plasmonic nanoparticles exhibit higher refractive index sensitivity due to large surface charge polarizability and local field enhancement.^3,4^ Strong response of LSPR peak position of anisotropic plasmonic nanoparticles enables the sensing of small molecules^5^ to large biomolecules.^6^ These nanoparticles can be utilized to construct LSPR based sensors both in solution and on the substrate. Due to difficulties in colloidal stability, immobilization of the particles on the substrate is gaining interest.^7^

Silver nanocubes (AgNCs) are anisotropic nanoparticles and are widely used in surface enhanced Raman scattering (SERS),^8–10^ fluorescence enhancement,^11–13^ refractive index based sensing^14–16^ due to its strong plasmonic properties. The synthesis of different edge-length nanocube strongly depends on the reaction atmosphere.^17^ Various routes have been followed to synthesize silver nanocubes including polyol synthesis,^17,18^ solvothermal method,^19–22^ hydrothermal synthesis,^23^ wet chemical method^24^ and microwave assisted method.^25–27^ In the synthesis process using ethylene glycol, silver nanocubes were synthesized by reducing the silver ions in ethylene glycol in the presence of polyvinylpyrrolidone (PVP) at higher temperatures. To promote the formation of perfect silver nanocubes chemical reagents such as HCl,^28,29^ Na_2_S,^30^ NaHS^31^ and FeCl_3_ ^32^ are used in the polyol process.

Formalin (37% aqueous solution of formaldehyde) is widely used for the increase of shelf-life of food products such as fish, meat and milk. Although, formaldehyde is also a metabolic product in animals, it is classified as human carcinogen and higher dose of it can cause eyes and nose irritation, damage to the central nervous system, immune system disorders, nasopharyngeal cancer and leukemia.^33^ The carcinogenic nature of formaldehyde makes its sensitive detection in the food product of utmost importance. In the recent past, several analytical methods such as high performance liquid chromatography^34^ (HPLC), gas chromatography^35,36^ (GC), gas chromatography/mass spectrometry^37,38^ (GC/MS), chemiluminescence^39,40^ and fluorimetry^41^ have been utilized to detect formaldehyde in trace amount. Although these methods show very high sensitivity for formaldehyde detection, the equipment used in these techniques are bulky and of high cost, which is not suitable for the on-site real time analysis of samples. Apart from these methods, among the optical detection modes, spectrophotometry^41–43^ and surface enhanced Raman scattering (SERS)^44–47^ have also been utilized for formaldehyde detection. While sensitivity of spectrophotometry method is limited, the equipment used in SERS is expansive. Also, SERS detection of formaldehyde requires derivatization of metal nanoparticles. Apart from these studies, silver nanoparticles sensitized titanium dioxide,^48^ Ag nanoparticle decorated carbon nanotube^49^ have also been used for formaldehyde sensing. Martínez-Aquino *et al.* have used resorcinol functionalized gold nanoparticles for the colorimetric detection of formaldehyde.^50^ Gold spherical nanoparticles and nanorods were also utilized for formaldehyde detection through refractive index sensing.^51^ Although, scattered reports on sensing of formaldehyde based on Ag and Au nanoparticle is available in literature, there is a need of further investigation of use of metal nanoparticles as formaldehyde sensor as these refractive index based sensors have potential to be developed into miniaturized and multiplexed platform.^52^

In this work, we present the synthesis of silver nanocubes (AgNCs) using PVP and sodium sulfide nonahydrate. The synthesis method used in the work is solvothermal method, as this method provides excellent control over the reaction atmosphere, which is necessary for the silver nanocube synthesis. The synthesized AgNCs of edge lengths 32 and 44 nm were investigated for their refractive index sensing capability utilizing the aqueous solution of glucose. The LSPR sensor chip is prepared by immobilization of the AgNCs on (3-aminopropyl)trimethoxysilane modified glass coverslips. The sensing capability of LSPR chip is also demonstrated with aqueous solution of glucose. In the final section of work, the sensitive detection of formaldehyde in water and diluted milk is demonstrated with 4-aminothiophenol functionalized synthesized Ag nanocubes.

## 2. Experimental

### 2.1 Chemicals and reagents

Chemicals used in the present work, silver nitrate (AgNO_3_, 99.5%), sodium sulfide nonahydrate (Na_2_S·9H_2_O, 98%), ethylene glycol (99.8%), 4-aminothiophenol (4-ATP, 97%) and (3-aminopropyl)trimethoxysilane (APTMS, 97%) were procured from Sigma-Aldrich. Polyvinylpyrrolidone (PVP) was purchased from Loba Chemie. Anhydrous glucose (99%) and ethanol (94–96%) were obtained from Alfa Aesar. All chemicals were used without any further purification. Deionized water from Milli-Q purification system was used for all synthesis.

### 2.2 Synthesis of Ag nanocubes

Silver nanocubes were synthesized by solvothermal method following the procedure reported in a previous report.^19^ For the synthesis, 100 μM of sodium sulfide (Na_2_S·9H_2_O) and 0.15 M PVP solution was prepared in 20 mL of ethylene glycol. This solution was mixed with 20 mL of 0.1 M AgNO_3_ in ethylene glycol with constant stirring. This mixture was transferred to 50 mL Teflon lined autoclave and heated for three hours. For three set of samples, the heating temperature was fixed at 145, 155 and 165 °C. After completion of the reaction, the autoclave was allowed to cool to room temperature naturally. The products were washed with acetone and water and centrifuged at 6000 rpm for 20 minutes. The particles settled at the bottom were re-dispersed in water and further used for characterizations and applications.

### 2.3 Spectral and morphological characterization

The spectral characterization of synthesized silver nanocubes was performed using lab-built UV-Vis spectroscopy setup explained in our earlier work.^53,54^ All the LSPR experiments were also performed with this lab-built experimental setup. The morphological characterization of synthesized silver nanocube was performed with FESEM. To record the FESEMS images, colloidal solution of silver nanocubes were drop casted on a gold coated glass coverslip. The images were acquired with TESCAN-MIRA 3 FESEM instrument.

### 2.4 Immobilization of Ag nanocubes on glass surface

The immobilization of AgNCs was performed on the silanized glass coverslips. Prior to silanization, the glass coverslip was cleaned in freshly prepared piranha solution (mixture of 3 : 1 conc. sulfuric acid to 30% hydrogen peroxide solution). Following the cleaning, the glass coverslips were rinsed vigorously with deionized water and dried. The dried coverslips were immersed in 10% APTMS ethanolic solution for fifteen minutes. Coverslips with APTMS as surface layer was then rinsed two times with ethanol and mildly sonicated in ethanol for one minute. Coverslips were further rinsed with deionized water followed by drying at 120 °C for three hours. The silanized coverslips were cooled to room temperature and immersed in silver nanocubes colloidal solution for different time span. The fabricated LSPR chip was stored in deionized water.

### 2.5 Functionalization of Ag nanocubes with 4-ATP

Functionalization of Ag nanocubes with 4-aminothiophenol (4-ATP) was performed for the detection of formaldehyde. For the functionalization, 0.5 mL of 10 mM 4-aminothiophenol solution in ethanol was added to 9.5 mL of Ag nanocubes colloidal solution and stored in dark. To ensure the functionalization, the solution mixture was centrifuged and the nanocubes were collected and re-dispersed in the deionized water.

## 3. Results and discussions

### 3.1 Synthesis and characterization of Ag nanocubes

**Fig. 1:**
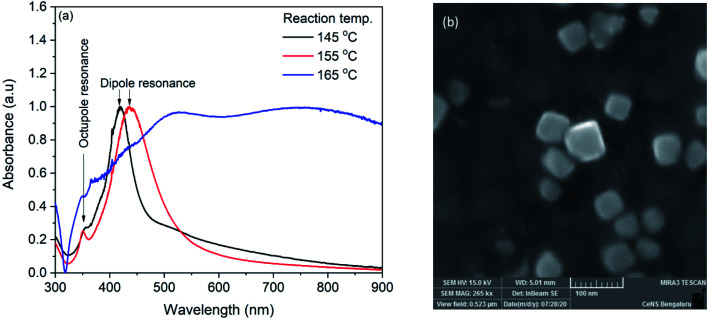
(a) UV-Vis spectra of silver nanocubes synthesized at 145, 155 and 165 °C (b) FESEM image of nanocube synthesized at 155 °C.

Silver nanocubes were synthesized by solvothermal method. In the synthesis, sodium sulfide was used with a fixed concentration of 50 μM. It has already been reported that the higher concentration of sodium sulfide (100 μM) leads to the formation of silver nanowires and lower concentration (12.5 μM) leads to the mixture of nanocubes and regular tetrahedrons.^19^ The reaction temperature was varied to tune the size of the nanocubes. The synthesized AgNCs were characterized with UV-Vis absorption spectroscopy with lab-built set up.^53,54^Fig. 1(a) shows the UV-Vis spectra of silver nanocubes synthesized at 145, 155 and 165 °C respectively. For the AgNCs obtained at 145 and 155 °C, the spectrum shows two distinct bands whereas a broad spectrum was observed for the product obtained at 165 °C. The broadness of this spectrum could be due to the aggregation of nanoparticles. The UV-Vis spectrum of AgNCs obtained at 145 °C shows a prominent band at ∼419 nm along with as shoulder band at ∼358 nm. For the AgNCs obtained at reaction temperature 155 °C, the prominent band was observed at 436 nm with a clear small band at 350 nm. The prominent band observed in two spectra are due to dipole resonance and the small band on lower wavelength side is due to the octupole resonance.^55^ The morphological characterization of synthesized AgNCs was performed with FESEM. Fig. 1(b) shows FESEM image of AgNCs synthesized at 155 °C reaction temperature. Although apart from AgNCs, some other shape nanoparticle can also be seen in the image, the average edge length of silver nanocubes obtained was found to be ∼44 nm. The nanocube synthesized at 145 °C shows ∼32 nm edge length.

### 3.2 Immobilization of silver nanocubes on glass substrate

**Fig. 2:**
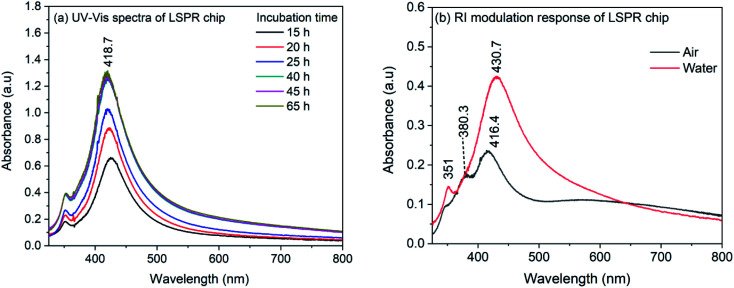
(a) UV-Vis spectra of AgNCs LSPR chip recorded for different incubation time (b) UV-Vis spectra of LSPR chip in air and in water.

The synthesized nanocubes were immobilized on glass coverslips to make an LSPR sensor chip. For the immobilization, the glass coverslips were silanized with APTMS and the silanized coverslips were incubated in the silver nanocube colloidal solution. The density of nanocubes on the coverslip surface was controlled by controlling the incubation time and is investigated by recording of UV-Vis spectrum. Fig. 2(a) shows the UV-Vis spectra of nanocubes on coverslips for different incubation time. As it can be seen, the spectrum shows both the characteristic bands of nanocube. Also, the spectrum do not show any change in intensity after 40 hours of incubation, indicating the saturation of the coverslip surface. It is also evident from the spectra that the full width at half maximum (FWHM) is smaller compared to the nanocube spectrum in the colloidal solution. For the colloidal solution, the FWHM was 100 nm which is reduced to ∼70 nm in case of immobilized nanocubes. The decrease in the FWHM could be due to the fact that in the immobilization process, nanocubes are aligned in the same plane with preferred orientation.^56^ It has to be noted that the absorption maximum does not show any red shift during the immobilization process which indicates that, although the nanocubes are immobilized on the APTMS modified glass surface, the inter-particle separation is higher compared to the edge-length of nanocubes^57^ and therefore, there is no plasmonic interaction between nanoparticles.

In order to ensure the sensitivity of the LSPR chip towards change in refractive index, the LSPR chip was dried in air and immersed in water. Fig. 2(b) shows the spectrum of dry LSPR chip and the effect of refractive index change from air to water (RI: 1.33). As it can be seen in the figure, the dried sensor chip showed bands at 416.4, ∼380 and 348 nm. The band at 416.4 nm corresponds to the dipolar mode whereas the 348 nm band is due to the octupolar resonance.^56^ It has been observed in earlier reports on AgNCs that the quadrupole resonance is dark mode and is absent in the colloidal nanocube solution. However, when nanocubes are immobilized and interaction occurs between nanocubes and substrate, this dark mode becomes active due to mode hybridization and marks it appearance on the blue-side of the dipolar band.^58–62^ The band appeared at 380 nm could be due to the quadrupolar mode appeared as a result of interaction of nanocube surface with the substrate. When the LSPR chip is immersed in water, the quadrupole resonance band disappeared completely and the dipolar resonance band shows a red-shift to 430.7 nm. It has also been observed that for small AgNCs, both dipole and quadrupole modes are sensitive to the RI change.^56^ So, it is possible that after the immersion of LSPR chip in water, the band representative of quadrupole mode merged with the dipolar band. However, a shift of 14.3 nm for the dipolar resonance band confirms the sensitivity of immobilized nanocubes on the APTMS modified glass substrate.

### 3.3 Refractive index sensitivity of Ag nanocubes towards glucose

**Fig. 3:**
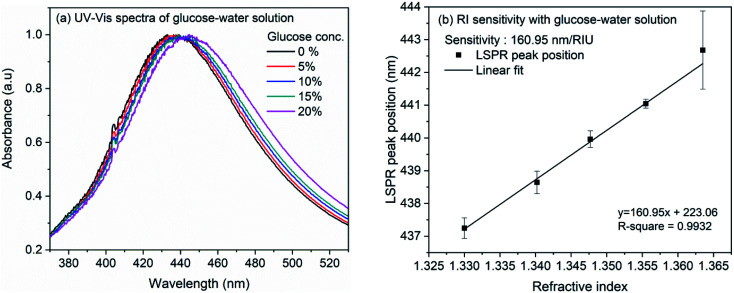
(a) UV-Vis spectra of silver nanocubes with glucose concentration from 0% to 20% (b) linearly fitted plot of LSPR band maxima *versus* refractive index of glucose solution.

The refractive index sensitivity of the synthesized AgNCs in colloidal solution was examined with aqueous solution of glucose. Although substantial amount of work have been reported on the detection of glucose, in the present work, we opted for this molecule as the refractive index of the solution can be controlled very minutely and accurately using this easily available water soluble small molecule. Therefore, rather than providing the sensitivity of nanocube towards glucose, we intend to evaluate the sensitivity of synthesized nanocubes in solution and on LSPR chip. For the experiment, glucose concentration was varied from 0 to 20% in the step of 5%. Fig. 3(a) shows the extinction spectra of AgNCs synthesized at 155 °C with variation of glucose concentration. The accurate position of LSPR bands were obtained by calculating the first order derivative of spectra near the LSPR band maxima. The numerical root of linear fit to the derivative data is considered as the LSPR band maximum position. Fig. 3(b) shows the variation of LSPR band maximum with refractive indices of glucose solution. The solid line in the figure represents the linear fit, the slope of which provides an estimate to the RI sensitivity. In case of AgNCs obtained at reaction temperature 155 °C, the sensitivity was 161 nm per RIU whereas the sensitivity of AgNCs obtained at 145 °C is 113.7 nm per RIU. The UV-Vis spectra and RI sensitivity plot for AgNCs obtained at 145 °C are shown in ESI Fig. S1(a) and (b).† Similar RI sensitivity of Ag nanocubes have been observed earlier reports. In solution, Elfassy *et al.* reported refractive index sensitivity of 125 nm per RIU for silver nanocubes of 50 nm side length^14^ whereas RI sensitivity of 158 nm per RIU was observed by Martinsson *et al.* for Ag nanocubes of 35 nm edge-length.^63^

**Fig. 4:**
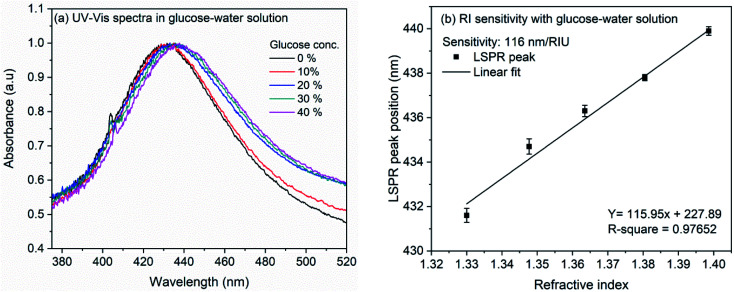
(a) UV-Vis spectra of LSPR chip showing the variation in LSPR peak position with increase in glucose concentration from 0% to 40% (b) linear fit of LSPR peak position *versus* refractive index of glucose in water.

The RI sensitivity of nanocubes was also measured on the LSPR chip prepared by immobilization of nanocubes on the glass coverslip. Fig. 4(a) shows the effect of various glucose concentrations on the extinction spectrum of nanocubes. The analysis (Fig. 4(b)) shows the bulk refractive index sensitivity of LSPR chip to be 116 nm per RIU. The observed RI sensitivity of nanocubes on substrate is lower compared to sensitivity in the colloidal solution. This observed reduction in the RI sensitivity could be due to the substrate. Several studies have been reported on the effect of substrate refractive index on the dipolar and quadrupolar plasmon resonance in the silver nanocubes.^56,59–64^ Mahmoud *et al.* observed refractive index sensitivity of 113 nm per RIU on the quartz (RI: 1.458) surface for 65 nm edge-length Ag nanocubes.^60^ Ahamad *et al.* observed that the refractive index sensitivity is reduced by ∼50% when Ag nanocube of side-length 40 nm was immobilized on the glass substrate. The sensitivity reduction was higher when nanocubes were immobilized on TiO_2_ thin layer.^56^ In the work by Ahamad *et al.*, refractive index sensitivity in solution was 176 nm per RIU which is reduced to 93 nm per RIU on glass and 57 nm per RIU on TiO_2_. In a similar study, Martinsson *et al.* observed low amount of reduction in the refractive index sensitivity when Ag nanocubes were immobilized on APTES or polyelectrolyte modified glass substrate.^63^ It was observed that, for the 40 nm edge-length Ag nanocubes, the sensitivity in solution was 158 nm per RIU which is reduced to 124 nm per RIU in case of APTES (RI: 1.420) modified glass substrate and up to 137 nm per RIU for polyelectrolyte film (RI: 1.46) modified glass substrate. All these studies indicate towards strong dependence of refractive index sensitivity towards the substrate. In the present work, the nanocubes were immobilized on APTMS (RI: 1.424) modified glass surface which is similar to the AgNCs immobilized on APTES modified surface and therefore the sensitivity reduction is consistent with earlier report.^63^

### 3.4 Formaldehyde detection using Ag nanocubes

#### 3.4.1 Functionalization of Ag nanocube with 4-ATP

**Fig. 5:**
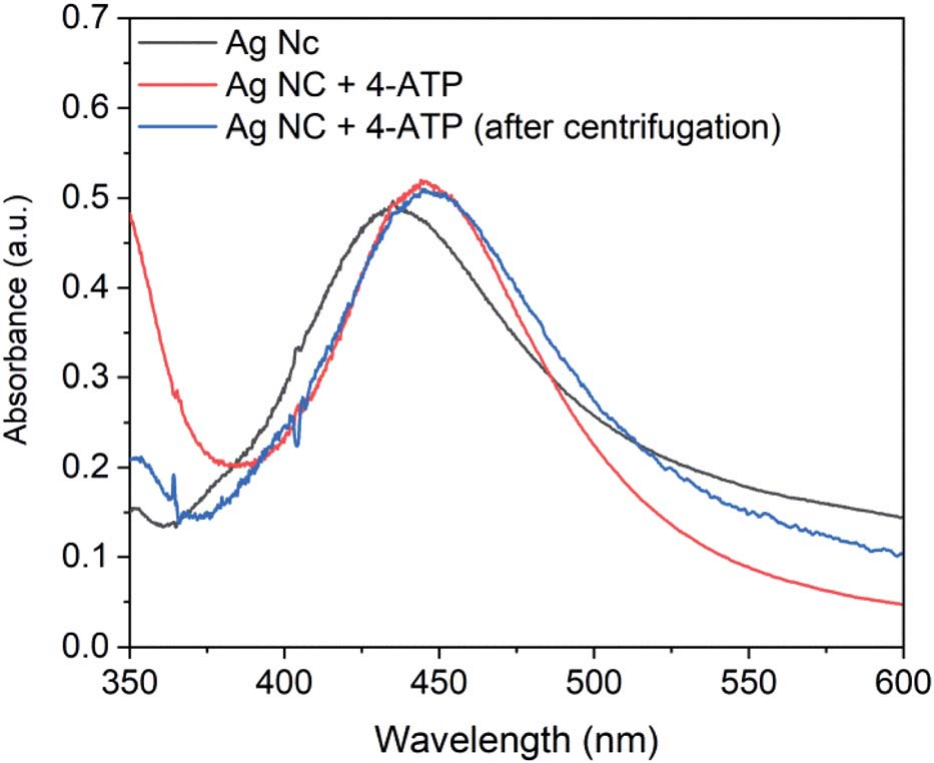
UV-Vis spectra of silver nanocubes, 4-ATP functionalized silver nanocubes and 4-ATP functionalized silver nanocubes after centrifugation.

Among the optical sensing technique, the colorimetric detection has been vastly utilized for the detection of formaldehyde. In these colorimetry based detection, basic reaction between nucleophilic aryl or alkyl amine with electrophilic carbon of formaldehyde was utilized.^65,66^ Following the same principle, in the present work, functionalization of synthesized nanocubes was performed with 4-aminothiophenol (4-ATP). For the functionalization, ethanolic solution of 4-ATP was mixed with colloidal silver nanocubes solution. The UV-Vis spectrum of silver nanocubes with 4-ATP was recorded and shown in Fig. 5 along with the spectrum of AgNCs without 4-ATP. As it can be seen in the figure, the dipolar resonance shifted from 437.5 to 451.4 nm. It has already been reported that the thiol group of 4-ATP strongly adsorbs to the surface of silver nanoparticles leaving the amine group free^67^ and the observed shift in spectrum could be a result of this binding. To ascertain that the observed spectral shift is a result of immobilization of 4-ATP on the AgNCs surface and not due to the refractive index variation in the AgNCs colloidal solution, the 4-ATP functionalized silver nanocubes solution was centrifuged and the nanoparticles were collected and re-dispersed in deionized water. The spectrum of the re-dispersed nanocubes is also shown in Fig. 5. Compared to the spectrum of AgNCs with 4-ATP, this spectrum does not show spectral shift indicating the immobilization of 4-ATP molecules on the surface of AgNCs.

It is important to mention that various concentration of 4-ATP from 0.5 mM to 2.0 mM was investigated for functionalization of nanocubes. However, it was observed that the concentration above 0.5 mM results in broader extinction spectrum. The broadness in the spectrum can result in poor resolution and therefore not adequate for sensing experiments. In view of this, the 4-ATP concentration for the functionalization of Ag nanocubes was kept constant at 0.5 mM.

#### 3.4.2 Formaldehyde sensing in water and milk

**Fig. 6:**
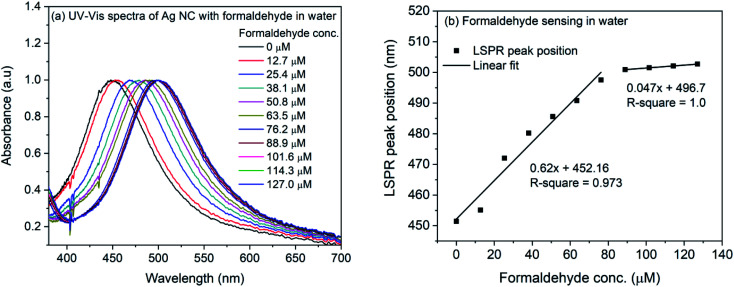
(a) UV-Vis spectra of 4-ATP functionalized silver nanocubes with different concentrations of formaldehyde in water (b) plot of LSPR peak position *vs.* concentration of formaldehyde in water.

Formaldehyde contaminated water and food products such as milk can cause serious health risk such as cancer in human beings. A tolerable concentration limit of 2.6 mg L^−1^ in ingested products has been recommended by World Health Organization (WHO).^68^ The severity of formaldehyde consumption through water or milk makes the detection of it very important in these two media. The 4-ATP functionalized silver nanocubes were utilized to sense formaldehyde in water and milk. For the sensing experiment in water, formaldehyde concentration was varied from 127 μM to 1270 μM (0.001% to 0.01% (v/v) of formalin in water). From the diluted formaldehyde solutions, 0.2 mL was mixed with 1.8 mL of 4-ATP functionalized silver nanocubes thus reducing the concentration of formaldehyde by a factor of ten. The mixture solution was incubated in dark for an hour followed by recording of UV-Vis spectra. Fig. 6(a) shows the UV-Vis spectra of the incubated samples. A progressive change in the LSPR band maximum can be seen in the figure. The band maxima position were plotted with the formaldehyde concentration and shown in Fig. 6(b). The solid line in the figure represents the linear fit to the data. It is evident that the response is linear below 76.2 μM concentration of formaldehyde with slope of 0.62 nm μM^−1^. Above this concentration, the saturation of sensitivity was observed with slope of 0.047 nm μM^−1^.

**Fig. 7:**
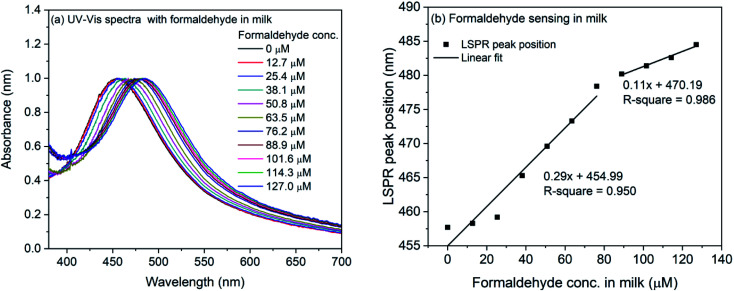
(a) UV-Vis spectra of 4-ATP functionalized silver nanocubes with different concentrations of formaldehyde in milk (b) plot of LSPR peak position *vs.* concentration of formaldehyde in milk.

The formaldehyde sensing experiment was also performed with commercially available milk sample. For the experiment, the milk was diluted and mixed with the 4-ATP functionalized silver nanocubes. The refractive index change due to milk was observed and the LSPR peak shifted from 451.4 nm to 457.7 nm. For the formaldehyde sensing, the diluted milk was contaminated with formaldehyde (final concentration: 127–1270 μM) and mixed with 4-ATP functionalized silver nanocubes followed by incubation for one hour in dark. The UV-Vis spectra of formaldehyde contaminated milk sample in 4-ATP immobilized nanocubes are shown in Fig. 7(a). As it is evident, successive change in the LSPR peak position occurs when different concentration of formaldehyde was used in the mixture solution. Fig. 7(b) shows the plot of LSPR band maxima with the concentration of formaldehyde in milk. Although the successive increase in the LSPR band position is observed in case of formaldehyde in milk samples, the extent of red-shift is nearly half compared to that for formaldehyde in water. This is also evident from the slope of linear fit in the lower concentration region. In case of milk, it is 0.29 compared to 0.62 in case of water. In case of water the observed red-shift was 51.3 nm for 0 to 1270 μM concentration of formaldehyde, whereas in case of milk samples, the observed shift is 26.8 nm.

The limit of detection LOD (3.3 × standard deviation of *y*-intercept/slope of regression line) and the limit of quantification LOQ (10 × standard deviation of *y*-intercept/slope of regression line) were calculated for formaldehyde in water and milk samples. The LOD and LOQ for formaldehyde in water was found to be 1.05 and 3.18 mg L^−1^. Whereas, in case of milk sample, the LOD and LOQ were 1.14 and 3.45 mg L^−1^ respectively.

**Fig. 8:**
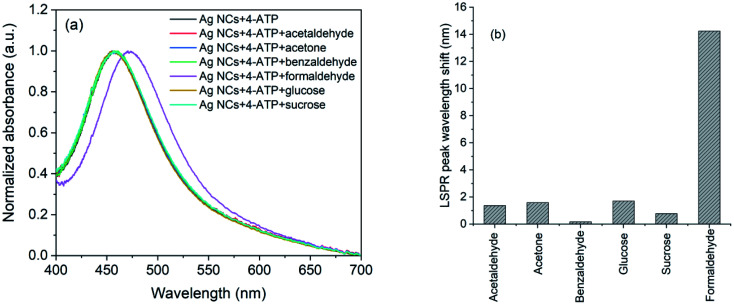
(a) UV-Vis spectra and (b) observed wavelength shifts for AgNCs–4-ATP with acetaldehyde, acetone, benzaldehyde, glucose, sucrose and formaldehyde.

The selectivity of the 4-ATP functionalized AgNCs towards formaldehyde *versus* acetaldehyde, benzaldehyde, acetone, glucose and sucrose were investigated following the same method. The concentrations of all the analyte molecules were 1 mM and the corresponding absorption spectra are shown in Fig. 8(a). As it is evident, the wavelength shift in presence of formaldehyde is very large compared to other analyte (Fig. 8(b)) which shows the selectivity of 4-ATP functionalized AgNCs towards formaldehyde.

**Scheme 1:**
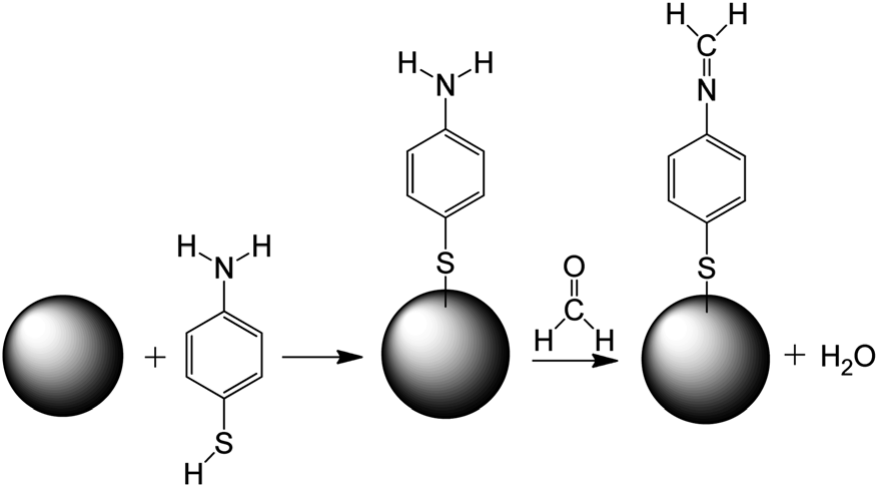
Mechanism of formaldehyde sensing using Ag nanocube (represented as sphere in the scheme) functionalized with 4-ATP.

The mechanism of sensing of formaldehyde with 4-ATP functionalized nanocubes can be understood from the Scheme 1 shown below. The Ag nanocube adsorbs the 4-ATP molecule on its surface though the thiol group. When this functionalized nanoparticle interacts with the formaldehyde molecule, the amine terminal of the 4-ATP molecule undergoes chemical modification to imine with a release of one water molecule. The chemical process seems to be irreversible in nature.

In some of the recent work, the formaldehyde sensing was performed with fiber optic sensor^69^ and silver nanocluster modified Tollen's reagent.^70^ Using the fiber optic sensor,^69^ lower detection up to 0.2 mg L^−1^ was achieved whereas the lower detection limit obtained in the method based on silver nanocluster is 27.99 μM (∼0.84 mg L^−1^).^70^ In the present work, the formaldehyde with concentration 12.7 μM (∼0.38 mg L^−1^) has been detected successfully. Similar to the earlier reported values, the sensitivity obtained in the present work is well below the tolerable limit (2.6 mg L^−1^) of formaldehyde in ingested food products. This establishes that the Ag nanocubes functionalized with 4-aminothiophenol molecules can be used for sensitive detection of formaldehyde in water and milk. The establishment of 4-ATP functionalized silver nanocube as formaldehyde sensor will also paves the way to use other Ag/Au nanoparticle for the formaldehyde detection with better sensitivity depending on their shape and size. Further, compared to other analytical methods, it will be easier to prepare LSPR sensor chips with these nanoparticles which can be extremely useful for the on-site field applications.

## 4. Conclusions

Silver nanocubes of edge-length 44 and 32 nm were synthesized by solvothermal method using PVP as capping agent in the presence of sodium sulphide. Colloidal suspension of silver nanocubes showed refractive index sensitivity of 161 nm per RIU for glucose solution in water. Silver nanocubes were immobilized on glass substrate using (3-aminopropyl)trimethoxysilane to construct LSPR chip. The refractive index sensitivity of the LSPR chip towards glucose was found to be 116 nm per RIU. The lower RI sensitivity could be due to the interaction of nanocubes with the substrate. Silver nanocubes were functionalized with 4-aminothiophenol for sensing of formaldehyde in water and milk. The sensitivity obtained for formaldehyde in water and in diluted milk are 0.62 nm μM^−1^ and 0.29 nm μM^−1^ respectively. Bulk refractive index sensitivity of silver nanocubes in solution and on the substrate proves the potential of it to be used in LSPR based sensing applications. Formaldehyde sensing capability of 4-aminothiophenol functionalized silver nanocubes demonstrates its usefulness in detecting adulterating molecules in milk.

## Conflicts of interest

Authors declare no conflict of interest.

## Supplementary Material

RA-011-D0RA10161C-s001
