# Supplementary material for: Refractive index and formaldehyde sensing with silver nanocubes
Source: RSC Adv. 2021 Feb 19;11(14):8042–50. doi: 10.1039/d0ra10161c (PMC8695105; doi:10.1039/d0ra10161c)
Supplement: RA-011-D0RA10161C-s001 [file RA-011-D0RA10161C-s001.pdf]

Electronic Supplementary Material (ESI) for RSC Advances.
This journal is © The Royal Society of Chemistry 2021

Refractive index and formaldehyde sensing with silver nanocubes

Hemant Ramakant Hedge, Santhosh Chidangil and Rajeev K. Sinha*

Department of Atomic and Molecular Physics, Manipal Academy of Higher Education, Manipal – 576104, Karnataka, India

*Email: rajeev.sinha@manipal.edu

Supplementary Materials

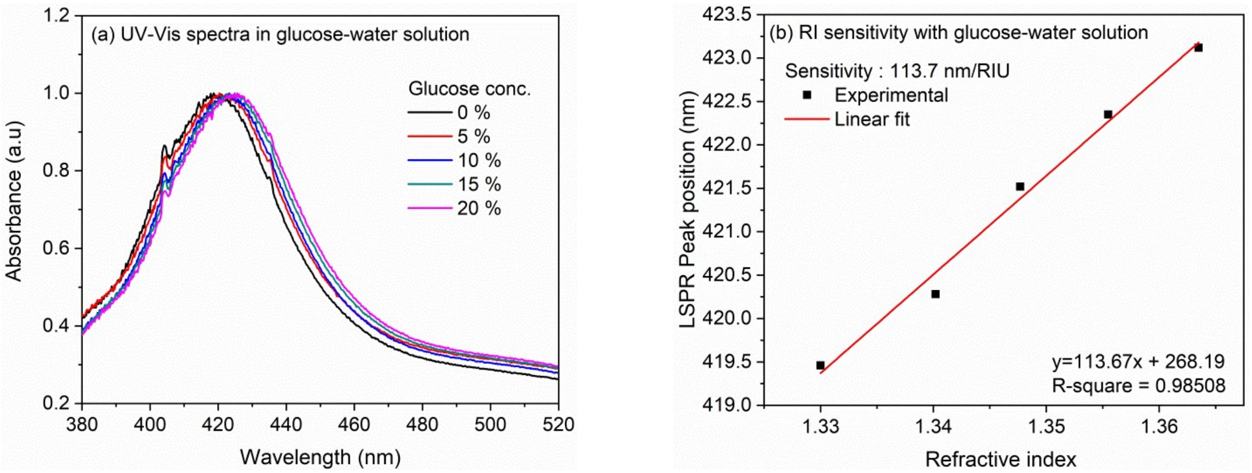

Figure S1. (a) UV-Vis spectra of silver nanocubes with glucose concentration from 0% to 20% (b) linearly fitted plot of LSPR band maxima versus refractive index of glucose solution. The 32 nm edge-length silver nanocubes were used in the study.
